# Spatial summation across the visual field in strabismic and anisometropic amblyopia

**DOI:** 10.1038/s41598-018-21620-6

**Published:** 2018-03-01

**Authors:** Shindy Je, Fergal A. Ennis, J. Margaret Woodhouse, Frank Sengpiel, Tony Redmond

**Affiliations:** 1 School of Optometry and Vision Sciences, Cardiff University, Cardiff, United Kingdom; 2 School of Biosciences, Cardiff University, Cardiff, United Kingdom

## Abstract

Ricco’s area (the largest area of visual space in which stimulus area and intensity are inversely proportional at threshold) has previously been hypothesised to be a result of centre/surround antagonism in retinal ganglion cell receptive fields, but recent evidence suggests a sizeable cortical contribution. Here, Ricco’s area was measured in amblyopia, a condition in which retinal receptive fields are normal, to better understand its physiological basis. Spatial summation functions were determined at 12 visual field locations in both eyes of 14 amblyopic adults and 15 normal-sighted controls. Ricco’s area was significantly larger in amblyopic eyes than in fellow non-amblyopic eyes. Compared to the size of Ricco’s area in control eyes, Ricco’s area measured significantly larger in amblyopic eyes. Additionally, Ricco’s area in the fellow, non-amblyopic eye of amblyopic participants measured significantly smaller than in control eyes. Compared to controls, Ricco’s area was larger in amblyopic eyes and smaller in fellow non-amblyopic eyes. Amblyopia type, binocularity, and inter-ocular difference in visual acuity were significantly associated with inter-ocular differences in Ricco’s area in amblyopes. The physiological basis for Ricco’s area is unlikely to be confined to the retina, but more likely representative of spatial summation at multiple sites along the visual pathway.

## Introduction

For a visual stimulus to be detected, the strength of the stimulus signal must overcome intrinsic noise that is inherent in the visual pathway. Pooling of signals over space (spatial summation) increases detectability, but at the expense of reduced visual resolution. Ricco’s law of spatial summation^1^ states that for a range of small stimulus areas, stimulus area (A) and intensity (I) are inversely proportional at threshold (A × I = k), i.e. spatial summation is complete. However, Ricco’s law applies only within a critical area, known as Ricco’s area. Beyond Ricco’s area, spatial summation is incomplete and, depending on the precise conditions under which it is measured, threshold is governed by laws of incomplete summation such as Piper’s law^2^ or Pieron’s law^3^.

The physiological basis for Ricco’s area is not entirely understood. The traditional explanation has been that Ricco’s area reflects spatial antagonism in retinal receptive fields (as has similarly been hypothesised by Westheimer^4^ as the basis for the critical area in sensitization functions), but more specifically, that it is the psychophysical correlate of the area of the retinal ganglion cell (RGC) receptive field centre^5,6^. Wilson^7^ noted that spatial summation functions across the visual field could be superimposed by a simple displacement along the area axis, and that threshold for the largest stimulus undergoing complete spatial summation was invariant across the visual field. This was attributed to differences in RGC receptive field overlap across the visual field, based on the correlation between RGC density and receptive field centre size^6,8^. Initially, it may seem reasonable that Ricco’s area has a retinal basis, given that it has also been found to vary with retinal eccentricity^7,9,10^ and background adaptation level^5,11^ in healthy observers. However, despite the close association between Ricco’s area and RGC dendritic field size^12^, as well as eccentricity-related changes in RGC density^9^, Pan & Swanson demonstrated that spatial summation of circular incremental stimuli, as used in clinical visual field testing, cannot be accounted for by probability summation across retinal ganglion cells, but by cortical pooling by multiple spatial mechanisms^13^. Further support for the hypothesis that cortical pooling contributes to the physiological basis of Ricco’s area comes from Redmond *et al*., who found changes in Ricco’s area in the S-cone pathway as a function of blue background adaptation level^14^. The traditional explanation that changes in Ricco’s area with background luminance occurs due to increased spatial antagonism in RGC receptive fields^5^ cannot account for the results reported by Redmond *et al*.^14^ because centre-surround spatial antagonism is not found in receptive fields of the small bistratified cells that mediate S-cone signal response. Rather, the blue/yellow ON and OFF receptive field regions are spatially coextensive^15^. Receptive fields of the arrangement S+/S− would be required to observe such changes, and these are not found at the level of the retina. Additionally, compared to its size in age-similar healthy controls, Ricco’s area was found to be larger in patients with glaucoma^14^, a disease characterized by the death of RGCs. The traditional concept of Ricco’s area as strictly a retinal phenomenon fails to reconcile the apparent shrinkage of RGCs^16,17^ with the documented enlargement of Ricco’s area in glaucoma. Other contributions to Ricco’s area, such as cortical pooling, may explain this structure-function discordance in glaucoma.

Since an enlarged Ricco’s area, such as occurs in glaucoma, can account for disproportionate deficits in contrast sensitivity to stimuli of different areas^14^, a better understanding of mechanisms other than retinal that contribute to Ricco’s area is essential to improve the design of functional visual field tests. The role of non-retinal contributions to Ricco’s area can be elucidated by determining whether a difference in Ricco’s area exists between eyes with normal vision and eyes with reduced vision in the absence of ocular or visual pathway pathology. Since amblyopia is a developmental disorder in which vision is reduced in the absence of detectable ocular or visual pathway disease, measuring spatial summation across the retina with stimuli of different areas in individuals with amblyopia may provide evidence for non-retinal contributions to Ricco’s area.

Approximately 3.6% of the UK population has amblyopia^18^. Histological studies of experimentally-induced amblyopia have suggested that the primary site of developmental neural deficit is V1^19–22^. RGCs have been observed to be anatomically and functionally normal (including normal spatial resolution^23^) in experimental models of amblyopia^24–26^. Although lateral geniculate nucleus (LGN) cells have been observed to change in size in severe deprivation amblyopia, their spatial resolution has been found to be unaffected^19,27–29^. Spatial acuity of X-cells in the LGN was also been found to be unaffected in cats with strabismic amblyopia^22^. Therefore, amblyopia is a suitable condition in which to investigate cortical contributions to Ricco’s area. Previous studies of spatial summation in amblyopia reported an accelerated rise in sensitivity with greater stimulus width in amblyopic eyes, reaching maximum sensitivity at much greater stimulus widths than in non-amblyopic eyes^30,31^, a finding that is suggestive of an enlargement of Ricco’s area in amblyopia.

The aim of this study was to form a better understanding of the physiological basis of Ricco’s area by investigating differences in spatial summation of perimetric stimuli between amblyopic adults and normally-sighted controls with binocular vision.

## Methods

Spatial summation functions were measured in both eyes of adults with strabismic or anisometropic amblyopia, and in normally-sighted controls with binocular single vision. Ricco’s area was estimated at each test location and analysed as a function of visual field eccentricity.

### Participants

Fourteen adults (median [IQR] age: 20.5 [19.25, 22.00] years) with amblyopia and 15 normally-sighted adults with normal binocular vision (median [IQR] age: 24 [22, 25] years) were recruited from staff and students of Cardiff University, as well as a research participant database at the Cardiff University Eye Clinic. All participants underwent an ophthalmic and orthoptic assessment, including a distance visual acuity test (Bailey-Lovie chart, logMAR notation), optical coherence tomography (Topcon 3D OCT 1000, Topcon Corp, Tokyo, Japan), and slit-lamp biomicroscopy with anterior eye assessment, to screen for any ocular or visual abnormalities that may otherwise affect visual performance. Binocular status was confirmed using tests for simultaneous perception (Bagolini lenses), suppression (Worth’s 4 dot test), stereopsis (TNO, Oculus, Wetzlar, Germany), eccentric fixation (ophthalmoscope grid), and the prism cover test. Binocular vision was confirmed if the participant demonstrated simultaneous perception, no suppression, and measurable stereopsis. Participants were included in the amblyopic group if they had an inter-ocular difference in visual acuity of ≥0.2 logMAR (two lines, or more, on the Bailey-Lovie chart). Anisometropic amblyopia was classified as an inter-ocular difference in refractive error of ≥1.00 DS, without strabismus or a history of strabismus surgery. Strabismic amblyopia was classified as amblyopia with a manifest strabismus, a history of childhood strabismus, or previous strabismus surgery. Each participant’s current distance refractive error was recorded or, if his/her refractive correction was >2 years old, a refraction was done as part of the research visit. Refractive correction, appropriate for the test distance of 33 cm, was worn during experiments. Appropriate refractive correction was also used for the relevant orthoptic assessments. Both eyes were included in the study.

Ethical approval was granted by the Wales Research Ethics Committee 1. Informed consent was obtained before participants were included. The research was conducted in accordance with the tenets of the Declaration of Helsinki.

### Apparatus and stimuli

**Figure 1:**
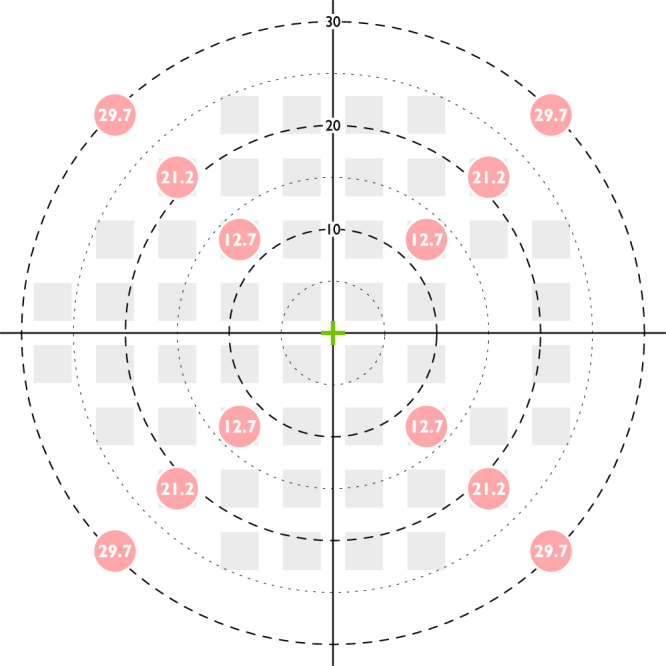
Visual field locations tested in the current study. A conventional 24-2 visual field pattern (right visual field), used in clinical visual field tests, is displayed for clinical reference.

An Octopus 900 perimeter (Haag Streit AG, Koeniz, Switzerland) was used to measure contrast thresholds by presenting circular achromatic luminance increments on an achromatic background of 10 cd/m^2^. Experiments were driven by the Open Perimetry Interface (OPI)^32^. Fixation was monitored visually, using the instrument’s fixation monitor. A 1:1 staircase and yes/no response criterion were used to determine individual thresholds. Presentation duration was 200 ms, with a square wave temporal profile. Stimuli were consecutively presented to 12 visual field locations (4 locations at each of 12.7°, 21.2°, and 29.7° eccentricity, Fig. 1).

### Procedure

Luminance contrast thresholds were measured for each of five Goldmann stimuli (I–V, having areas of 0.01, 0.04, 0.15, 0.58, and 2.27 deg^2^, respectively) at each of the 12 locations, one area at a time (randomly ordered). After thresholds for all five stimulus areas were determined for all 12 locations, the entire procedure was repeated. For each of the 12 locations and for each stimulus area, the two threshold measurements were averaged. Monocular thresholds were measured separately for each eye, with eye order randomized between participants.

### Statistical analysis

For each of the three eccentricities, the four threshold measurements (one per quadrant) for each stimulus size were averaged to give one spatial summation function at each eccentricity. In order to estimate Ricco’s area at each eccentricity, an iterative two-phase regression analysis^33^ was performed on the eccentricity-averaged data. The fitting procedure is described in depth in our previous work^34^; briefly, a two-phase regression function was fitted to the threshold data for the five stimulus areas, constraining the slope of the first line to −1 (respecting Ricco’s law), and allowing the intercept of the first line, the slope of the second line, and the breakpoint value to vary. The breakpoint, estimated by the model, was designated as Ricco’s area. Each eye had three Ricco’s area estimates, one for each eccentricity.

In the amblyopic participants, Ricco’s area estimates were compared between amblyopic and non-amblyopic eyes at each eccentricity. Similarly, Ricco’s area estimates were compared between the right and left eyes of normal participants. Inter-ocular differences were tested at each eccentricity with a paired t-test. A Holm-Bonferroni correction was applied to p-values obtained for each of the three eccentricities.

To investigate the effect of (a) type of amblyopia, (b) binocularity, and (c) inter-ocular difference in central visual acuity on the difference in Ricco’s area between amblyopic and fellow non-amblyopic eyes, linear mixed effects model analysis was performed, with the inter-ocular difference in Ricco’s area as the dependent variable, and type of amblyopia, binocularity, and inter-ocular difference in VA as fixed effects. Participant and eccentricity were included as random effects, with random intercepts added to the by-participant and by-eccentricity effects. For this analysis, data from each eccentricity and from each participant were pooled into a single dataset. The magnitude of each of the effects was determined from the analysis. Likelihood tests of the model (including all effects) and of the same model with the effect in question removed, were performed in order to determine the statistical significance of that effect.

Fixation has been found to be more unstable in amblyopic eyes than in those of normal controls^35^. Fixation stability was not measured prospectively in this study, but the choice of test locations vertically and horizontally symmetrical about the fixation point, each tested in an interleaved fashion, was made in an attempt to avoid fixation drift in a particular direction. Therefore, any fixation instability is more likely of the form that would contribute increased variance in the measurement of Ricco’s area rather than a systematic bias towards larger or smaller sizes. If Ricco’s area encompasses a constant number of RGCs at different locations across the visual field^36^, the greatest variance in its measurement is expected to be found in regions of the visual field where the underlying RGC density gradient is steepest. The likely limits of variance in the measurement of Ricco’s area was calculated at the most central visual field eccentricity (12.7°) for the amblyopic participant with the worst visual acuity (i.e. the ‘worst case scenario’). First, eccentricity was converted from degrees to millimetres on the retina^37^. Mean RGC/mm^2^ at this eccentricity was then determined from the study of Curcio & Allen^38^. The relationship between visual acuity and fixation instability (the area of a bivariate contour ellipse encompassing fixation locations, BCEA) was determined from Chung *et al*.^35^. The predicted BCEA for our participant with the worst visual acuity was then determined. Assuming that the bivariate contour forms a circle rather than an ellipse (a necessary assumption in order to obtain a value for the extent of the region), the radius (in mm) was calculated and used to calculate the predicted limits of deviation from the intended test location, both proximal and distal to the fovea. The expected difference in RGC density between the intended test location and these limits was determined from Curcio & Allen^38^. Assuming a constant number of RGCs underlying Ricco’s area at any given location in the visual field^36^, the expected limits of variance in the measurement of Ricco’s area were calculated.

Statistical analysis was performed with the open source statistical environment R^39^, and the lme4 package^40^, where applicable.

The datasets generated and analysed during the current study are available from the corresponding author on reasonable request.

## Results

Clinical characteristics of amblyopic participants are outlined in Table 1 and those of control participants are outlined in Table 2. Seven of the amblyopic participants had strabismic amblyopia (of which three had binocular vision), while the remaining seven had anisometropic amblyopia (of which four had binocular vision). Visual acuity was, on average, 0.42 logMAR (approx. 4 lines) lower in the amblyopic eye than in the fellow non-amblyopic eye (paired t-test, p < 0.001). Visual acuity in the non-amblyopic eye was, on average, 0.04 logMAR (2 letters) better than the average visual acuity for the right and left eyes in the control cohort, but this was not statistically significant (Student’s t-test, p = 0.12).

**Table 1:** Clinical characteristics of amblyopic participants in the current study.

Participant ID (age, years) Type of Amblyopia	Refractive error	Visual Acuity (LogMAR)	Stereoacuity	History
A1 (18) Strabismic	R + 4.50/−1.50 × 50	−0.10	Absent	RE 2∆ Esotropia, 3∆ Hypertropia, Patched and spectacles @ 3yrs, No surgery
	L + 4.50/−1.50 × 15	−0.30		
A2 (23) Strabismic	R −4.25/−0.75 × 100	−0.08	Absent	LE 8∆ Esotropia, Patched and spectacles @ 5yrs, No surgery
	L −3.75/−0.50 × 82	0.32		
A3 (19) Strabismic	R + 1.00/−0.50 × 100	−0.10	200 sec arc	LE Esotropia noticed at 5 yrs, Patched and spectacles @ 5 yrs, Surgery @ 5 yrs.
	L + 1.50/−0.50 × 65	0.12		
A4 (21) Strabismic	R + 7.25DS	0.36	Absent	RE 6∆ Esotopia, Patched and spectacles @ 2 yrs.
	L + 6.75/−0.25 × 170	−0.16		
A5 (27) Strabismic	R + 1.25/−1.50 × 155	−0.10	Absent	LE 6∆ Esotropia, Patched and spectacles @ 2 yrs.
	L + 1.25/−0.75 × 55	0.20		
A6 (22) Strabismic	R + 1.25/−0.50 × 90	0.16	400 sec arc	Microtropia, Not patched, No surgery.
	L + 1.00/−0.25 × 130	−0.22		
A7 (22) Strabismic	R + 0.75/−0.50 × 180	0.30	400 sec arc	LE 16∆ Hypotropia, Not patched, No surgery.
	L + 1.00/−0.75 × 173	−0.12		
A8 (35) Anisometropic	R + 6.00/−1.50 × 180	0.86	Absent	No manifest deviation, Patched and spectacles @ 7 yrs, No surgery.
	L + 0.50DS	−0.26		
A9 (21) Anisometropic	R + 5.00/−0.50 × 30	0.20	Absent	No manifest deviation, Patched and spectacles @ 4 yrs.
	L + 0.75/−0.25 × 120	−0.20		
A10 (20) Anisometropic	R + 2.25/−2.75 × 3	−0.14	400 sec arc	No manifest deviation, Patched and spectacles @ 3 yrs, No Surgery.
	L + 5.00/−3.75 × 171	0.36		
A11 (20) Anisometropic	R + 2.50/−0.25 × 180	0.62	Absent	No manifest deviation, Patched and spectacles @ 6 yrs, No surgery.
	L Plano	−0.10		
A12 (18) Anisometropic	R + 3.50/−1.50 × 180	0.10	240 sec arc	No manifest deviation, Patched and spectacles @ 6 yrs, No surgery.
	L + 1.25/−0.25 × 180	−0.10		
A13 (20) Anisometropic	R + 1.50/−4.50 × 28	−0.10	200 sec arc	No manifest deviation, Optical penalization @ 4 yrs, No surgery.
	L −0.75/−1.25 × 142	0.16		
A14 (19) Anisometropic	R + 0.50/−0.25 × 90	−0.20	400 sec arc	No manifest deviation, Not patched, Spectacles @ 8 yrs, No surgery.
	L + 2.75/−0.25 × 10	0.00

**Table 2:** Clinical characteristics of control participants in the current study.

Participant ID (age, years)	Refractive error	Visual Acuity (LogMAR)	Stereoacuity	History
C1 (29)	R Plano	−0.10	40 sec arc	No binocular vision anomalies
	L Plano	−0.10		
C2 (25)	R −5.50DS	−0.20	40 sec arc	No binocular vision anomalies
	L −4.50DS	−0.20		
C3 (24)	R −2.50/−1.00 × 175	−0.20	40 sec arc	No binocular vision anomalies
	L −3.00/−0.75 × 180	−0.20		
C4 (32)	R −1.50/−2.50 × 82	−0.10	40 sec arc	No binocular vision anomalies
	L −2.50/−2.00 × 81	−0.10		
C5 (21)	R −5.00/−0.50 × 120	−0.20	40 sec arc	No binocular vision anomalies
	L −5.00/−0.25 × 45	−0.20		
C6 (25)	R Plano	−0.20	40 sec arc	No binocular vision anomalies
	L Plano	−0.20		
C7 (25)	R Plano	−0.10	40 sec arc	No binocular vision anomalies
	L Plano	−0.10		
C8 (48)	R Plano	−0.20	40 sec arc	No binocular vision anomalies
	L Plano	−0.20		
C9 (24)	R + 0.50DS	−0.20	40 sec arc	No binocular vision anomalies
	L + 0.50DS	−0.20		
C10 (22)	R + 0.50/−0.25 × 160	−0.22	60 sec arc	No binocular vision anomalies
	L + 0.50/−0.25 × 180	−0.22		
C11 (23)	R −0.75/−0.25 × 180	−0.16	40 sec arc	No binocular vision anomalies
	L −0.50/−0.25 × 180	−0.22		
C12 (25)	R −0.25DS	−0.30	40 sec arc	No binocular vision anomalies
	L Plano	−0.30		
C13 (22)	R −0.25/−0.75 × 80	−0.20	40 sec arc	No binocular vision anomalies
	L −0.25/−0.50 × 55	−0.20		
C14 (19)	R + 0.25/−0.25 × 180	−0.24	40 sec arc	No binocular vision anomalies
	L + 0.25/−0.25 × 180	−0.28		
C15 (21)	R + 0.25DS	−0.24	40 sec arc	No binocular vision anomalies
	L Plano	−0.22		

**Figure 2:**
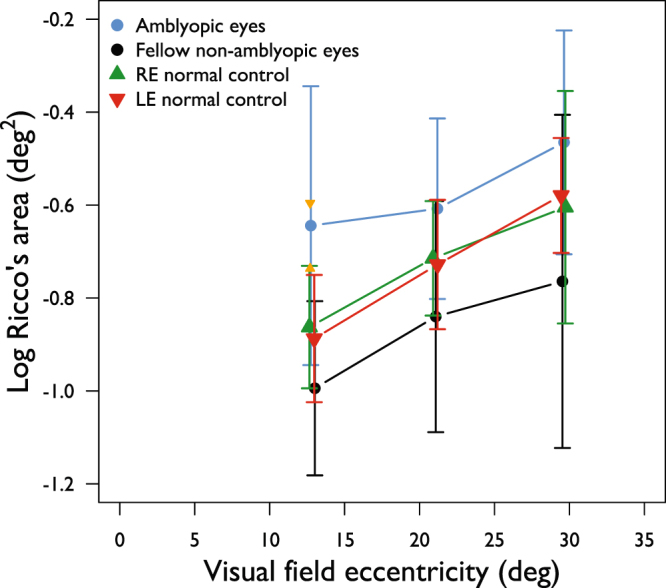
Ricco’s area as a function of visual field eccentricity in amblyopic and fellow non-amblyopic eyes (blue, black discs respectively), as well as the left and right eye of a normal control cohort (red, green triangles respectively). Jitter has been added to the x-values to aid visualisation of the data. Orange triangles represent the predicted limits of variance in the measurement of Ricco’s area due to fixation instability at an eccentricity of 12.7° (see main text for a full explanation). Error bars: SD.

A total of 174 spatial summation functions (3 eccentricities in 58 eyes) were determined across both groups. Figure 2 shows average Ricco’s area as a function of visual field eccentricity for amblyopic eyes, for non-amblyopic eyes of the same participants, and for left and right eyes of control participants. Our data show that Ricco’s area is larger at more peripheral test locations, as reported in previous literature^7,9,10^. At all eccentricities, mean Ricco’s area is larger in the amblyopic eyes, than in the fellow non-amblyopic eyes (all p < 0.01). Inter-ocular differences in Ricco’s area in the control group were negligible (Fig. 2). With this in mind, and given that there is no reasonable reason to suspect inter-ocular differences in Ricco’s area at corresponding visual field locations in normally-sighted observers, values for the right and left eyes were averaged at each eccentricity for further analysis, in order to reduce variance in the control data. Mean Ricco’s area is larger in amblyopic eyes, and smaller in the fellow non-amblyopic eyes, than in normal eyes at each eccentricity (Fig. 2 and Table 3). The difference in mean Ricco’s area between amblyopic eyes and those of normal controls is only statistically significant at 12.7° eccentricity (p = 0.047, following a Holm-Bonferroni correction). The orange triangles in Fig. 2 demonstrate the predicted limits of variance in the measurement of Ricco’s area for the eye with the worst visual acuity (0.86 logMAR) and at the test eccentricity with the steepest RGC density gradient (12.7° eccentricity). For illustration purposes, these limits are plotted around the mean Ricco’s area for that eccentricity. Given that this was the ‘worst case scenario’, variance would be expected to be less at all other test locations in all other participants.

**Table 3:** Difference in mean Ricco’s area between amblyopic participants and normal controls (normal Ricco’s area averaged by eye).

Eccentricity (deg)	Amblyopic eyes (deg^2^)	p-value (Student’s t-test)	Fellow non-amblyopic eyes (deg^2^)	p-value (Student’s t-test)
12.7	0.35	0.047*	−0.12	0.166
21.2	0.11	0.125	−0.13	0.255
29.7	0.13	0.125	−0.18	0.255

*Statistically significant at 0.05 level (Student’s t-test, Holm-Bonferroni-corrected p-values).

In subsequent analyses involving linear mixed effects models, inspection of the residuals confirmed normality and no heteroscedasticity.

### Binocular vs non-binocular vision in amblyopes

**Figure 3:**
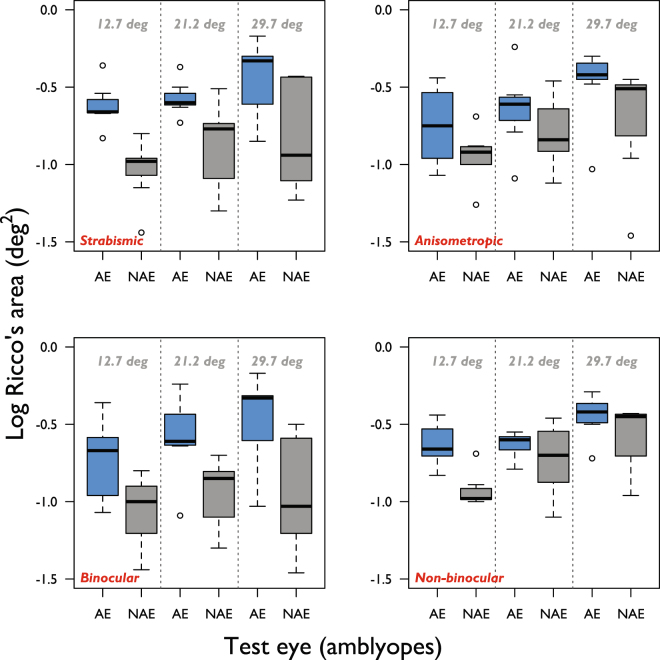
Ricco’s area in amblyopic (blue boxes) and non-amblyopic (grey boxes) eyes at each eccentricity when amblyopes are separated into strabismic (top left) and anisometropic (top right) groups, as well as binocular (bottom left) and non-binocular (bottom right) groups.

**Figure 4:**
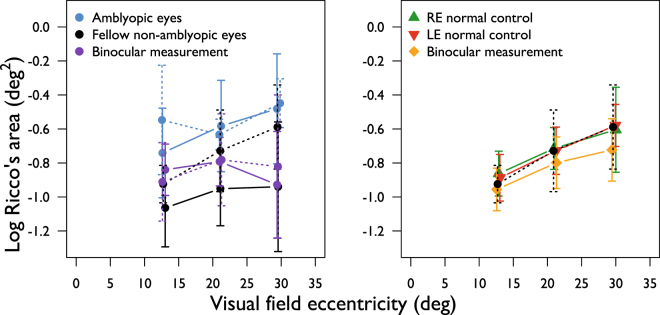
Mean Ricco’s area as a function of eccentricity in amblyopic participants, separated by binocular and non-binocular status (solid and dotted lines respectively, left panel). Mean Ricco’s area as a function of eccentricity in normal controls are shown in the right panel. For ease of comparison, Ricco’s area data from the fellow non-amblyopic eyes of non-binocular amblyopes are superimposed on data from normal controls (right panel, black symbols). Jitter has been added to the x-values to aid visualisation of the data. Error bars: SD.

Data were separated according to binocular and non-binocular vision status. Distributions of Ricco’s area values for the amblyopic and non-amblyopic eyes of these two groups can be seen in Fig. 3. Linear mixed effects model analysis reports that the inter-ocular difference in Ricco’s area is 0.33 log deg^2^ (±0.1 SE) larger overall in the binocular group than in the non-binocular group (p = 0.005). Separate linear mixed effects analyses, with Ricco’s area in the amblyopic and non-amblyopic eyes as dependent variables, reveals that most of this effect can be attributed to a smaller Ricco’s area in the non-amblyopic eyes of binocular, compared to non-binocular, amblyopes (by −0.327 log deg^2^, ±0.1 SE, p = 0.004). This difference can be seen in Fig. 4 (left panel; solid lines: binocular group, dotted lines: non-binocular group). In fact, mean Ricco’s area in the non-amblyopic eye of non-binocular amblyopes is comparable to that in normal controls at each eccentricity. The difference in Ricco’s area in the amblyopic eye between binocular and non-binocular groups was negligible (−0.003 log deg^2^, ±0.1 SE, p = 0.96).

### Strabismic vs anisometropic amblyopia

None of the strabismic participants in the study had anisometropia (Table 1). The distributions of Ricco’s area values for the amblyopic and non-amblyopic eyes of strabismic and anisometropic amblyopes are shown in Fig. 3. Linear mixed effects model analysis shows that type of amblyopia was significantly associated with the inter-ocular difference in Ricco’s area (p = 0.01). The inter-ocular difference in Ricco’s area was 0.28 log deg^2^ larger (±0.1 SE) in strabismic amblyopes than in anisometropic amblyopes. A smaller Ricco’s area in the non-amblyopic eyes of strabismic amblyopes relative to that in the non‐amblyopic eyes of anisometropic amblyopes (by −0.20 log deg^2^, ±0.1 SE, p = 0.06) contributed most to this effect. A slightly larger Ricco’s area in amblyopic eyes of strabismic amblyopes relative to that in amblyopic eyes of anisometropic amblyopes contributed to the effect by a negligible amount (+0.08 log deg^2^, ±0.1 SE, p = 0.40).

### Severity of amblyopia

The inter-ocular difference in central visual acuity to standard optotypes is taken as a clinical measure of severity of amblyopia. The linear mixed effects model shows that inter-ocular difference in VA is significantly associated with the inter-ocular difference in Ricco’s area (p = 0.04).

### Monocular vs binocular measurements of Ricco’s area

**Figure 5:**
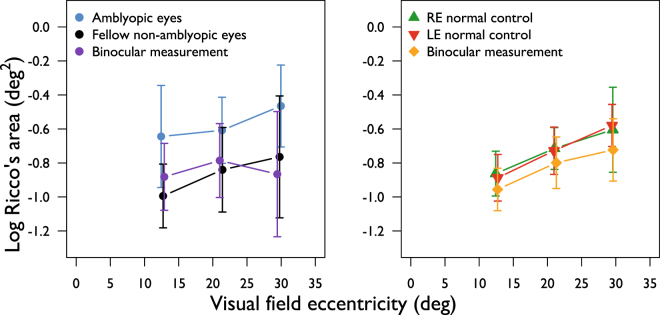
Mean Ricco’s area, measured binocularly, in amblyopic participants (purple discs) and normal controls (gold triangles) in left and right panels respectively. Mean Ricco’s area estimates in amblyopes and normal controls are also shown, for reference. Jitter is added to the x-values to aid visualisation of the data. Error bars: SD.

A subset of amblyopic participants (n = 12) and all controls (n = 15) underwent binocular measurements of Ricco’s area with an identical test protocol to that described in the Methods, in an attempt to understand if the anomalies of Ricco’s area found in the monocular experiments translate to binocular viewing, or if they are eliminated by binocular summation. In controls, Ricco’s area estimates measured binocularly are smaller, on average, than those measured monocularly at all eccentricities, with the largest difference in mean Ricco’s area observed at 29.7° (Fig. 5). None of these differences are statistically significant, however (all p ≥ 0.065; one-tailed paired t-test with Holm-Bonferroni correction). In amblyopes, binocularly measured Ricco’s area was, on average, smaller than that measured monocularly at all eccentricities in amblyopic eyes, reaching statistical significance only at 29.7° eccentricity (12.7°, p = 0.06; 21.2°, p = 0.06; 29.7°, p = 0.009; one-tailed paired t-test with Holm-Bonferroni correction). Binocular Ricco’s area was larger than that measured monocularly in fellow non-amblyopic eyes, at 12.7° and 21.2° (Fig. 5), but smaller than the monocular measurement at 29.7° eccentricity. None of the differences between binocular Ricco’s area and that measured in the fellow non-amblyopic eye were statistically significant (all p ≥ 0.22; one-tailed paired t-test with Holm-Bonferroni correction). Mean Ricco’s area, measured binocularly was, on average, larger at 12.7° and smaller at 29.7° eccentricity in amblyopic eyes than in control eyes, with a negligible difference at 21.2° eccentricity (all p ≥ 0.35; one-tailed t-test with Holm-Bonferroni correction). When data were separated by binocular vision status, mean binocularly measured Ricco’s area was comparable between groups at 21.2° eccentricity. On average, binocularly measured Ricco’s area was slightly smaller in the non-binocular than in binocular amblyopes at 12.7°, while the opposite was found at 29.7° (all p > 0.5; one-tailed t-test with Holm-Bonferroni correction; Fig. 4, purple symbols).

## Discussion

This study is the first to formally report on measurements of Ricco’s area in amblyopic participants. The finding of a larger mean Ricco’s area in amblyopic eyes, suggesting greater spatial summation than in normal eyes, supports previous findings of disproportionately higher thresholds for small stimuli than for larger stimuli in amblyopic cohorts^30,41,42^. Intriguingly, Ricco’s area is not only larger in amblyopic eyes than in control eyes, but also smaller (particularly for non-binocular participants) in the fellow non-amblyopic eyes of amblyopic participants than in eyes of normally-sighted control participants.

The results of this study offer insight to potential loci of visual pathway contributions that establish Ricco’s area. RGCs and the LGN previously have been reported as normal in amblyopia, including in form deprivation and strabismic amblyopia^19,22,25,27–29^. Also, research generally has shown that retinal nerve fibre layer thickness is unaffected in the condition^43–46^. Therefore, the findings of our study support predictions that Ricco’s area is not solely a retinal phenomenon, but that it likely represents summation by multiple mechanisms along the visual pathway^13^, i.e. a ‘net’ receptive field, or ‘perceptive field’^12,47^ for a given location in visual space. Indeed, differences in spatial summation in pathological conditions affecting the visual pathway from the retina to visual cortex^48,49^, as well as under changing S-cone adaptation conditions^14^, also support this concept of multiple mechanisms contributing to the extent of Ricco’s area.

Our results suggest either a shift in signal processing to the most responsive spatial frequency channels (as has been hypothesized to occur in glaucoma^48^), or a difference in neural convergence from lower level neurons to higher level neurons between amblyopic and non-amblyopic eyes. While both are plausible explanations, it is worth considering the finding of a smaller Ricco’s area in fellow non-amblyopic eyes. It is difficult to understand how the visual system may gain both from a shift to a channel tuned to lower spatial frequencies in the amblyopic eye and a shift to a channel tuned to higher spatial frequencies in the fellow non-amblyopic eye. Such an adaptation would suggest that the amblyopic eye is optimized for contrast sensitivity, and the fellow eye optimized for visual resolution. Alternatively, if receptive fields of retinal and LGN cells and the LGN are unaffected in amblyopia and the number of RGCs is similar between amblyopic eyes and fellow non-amblyopic eyes (as denoted by a lack of any notable difference in retinal nerve fibre layer thickness in published literature), the first site at which disrupted circuitry, and thus an anomaly of spatial summation, might occur is layer 4 of V1. One possible theory to explain our findings is that Ricco’s area could be influenced by anomalies of signal convergence at the level of the ocular dominance (OD) columns in amblyopia. OD asymmetry has been noted in primates^50,51^ and humans with early-onset deprivation amblyopia^52^, as well as an asymmetry in population receptive field size^53^. In contrast, ocular dominance columns relating to the right and left eyes of normally-sighted controls in a given hypercolumn are equal in width and contain comparable numbers of cells sampling the visual field. Therefore, in those eyes, 1 minute of visual angle is represented by the same cortical space when viewing with the right or left eye. Likewise, the number of geniculocortical axons relaying right eye signals from the LGN to layer 4 is equal to the number of geniculocortical axons relaying left eye signals. In amblyopia, the region of the hypercolumn sampling the visual field of the non-amblyopic eye is larger (wider OD columns), and the region of that sampling the visual field of the amblyopic eye is smaller (narrower OD columns). Therefore, 1 minute of visual angle, viewed through the fellow non-amblyopic eye would be represented by a larger area of cortex than the same visual angle viewed with the amblyopic eye, despite no difference in object size. Importantly, the proportions of geniculocortical axons relaying eye-specific signals from the LGN remain unaffected. Increased axonal arbor complexity in the geniculocortical cells mediating the signal response of the non-amblyopic eye, as seen in monocularly-deprived cats^54^, would mean that those axons are available to synapse with a greater number of cells in the hypercolumn, while reduced axonal arbor complexity in geniculocortical cells mediating the signal response from the amblyopic eye^54^ would mean that fewer cells in V1 will synapse with them. Assuming that the density of cells in OD columns is unaffected in amblyopia, greater spatial summation might therefore be observed as signals from the amblyopic eye converge on to a smaller region of the cortex (smaller number of cells), and vice versa. It should be noted that data on differences in OD column thickness and geniculocortical axon arbor complexity in mild amblyopia are unavailable; therefore our proposed theory is speculative, based on the assumption of a continuum of these effects from mild to severe amblyopia. Support for this assumption comes from a study on strabismic and anisometropic amblyopic monkeys that found the relative widths of the OD columns representing the amblyopic eye were reduced in proportion to the age of onset and the duration of the early visual abnormality; moreover the reduction in contrast sensitivity was in line with the reduction in relative OD column width^51^. Confirmation of such an explanation would require further study. A third, alternative explanation for an enlarged Ricco’s area in amblyopic eyes may be increased topographical disarray in receptive fields of V1 to at least V3, as reported in a recent fMRI study of amblyopia^53^.

The finding of a smaller Ricco’s area in fellow non-amblyopic eyes was unexpected, and so peripheral resolution acuity was not prospectively measured. Given the inverse association between spatial summation and resolution acuity, however, this finding suggests that resolution acuity should be higher in the non-amblyopic eye than in normal controls, albeit at the expense of spatial pooling. Conventionally, the non-amblyopic eye has been referred to as ‘the normal eye’ by clinicians due to its largely unaffected performance in visual acuity tasks on a high contrast letter chart. However, published evidence of the normality of visual performance of fellow non-amblyopic eyes is, as yet, inconclusive^55–60^. McKee *et al*.^55^ reported superior contrast sensitivity in non-amblyopic eyes of participants with a visual acuity of 6/30 or better in the amblyopic eye, but this superiority is only observed in participants without residual binocular function. Numerous studies comparing the visual function of the fellow non-amblyopic eye have, however, reported impairment in several attributes of visual function, such as contrast sensitivity^60,61^, Vernier acuity^62,63^, global motion processing^64^, dark adaptation^65^, rarebit sensitivity^66^, and an increase in neural noise^67^. Standard optotype visual acuity, measured at the fovea in this study, was not significantly different between fellow non-amblyopic eyes and those of normal controls. Although these measurements were not performed at the same test locations as measurements of Ricco’s area, the results of the current study indicate that non-amblyopic eyes would otherwise be inappropriately considered ‘normal’ in the clinical setting, despite a possible anomaly of Ricco’s area. A formal investigation of peripheral grating resolution acuity at the same test locations in those eyes is warranted.

In this study, the findings of a larger-than-normal Ricco’s area in amblyopia and a smaller-than-normal area in the fellow eye were in a cohort containing an equal proportion of anisometropic and strabismic amblyopes. Binocular and non-binocular amblyopes were also represented in equal proportions, with strabismic and anisometropic amblyopes represented among both groups. Statistical analysis indicated that binocularity had the largest effect on inter-ocular differences in Ricco’s area in amblyopic participants. While the difference in mean Ricco’s area between the two binocularity groups for amblyopic eyes was negligible, most of the effect of binocularity could be explained by a smaller Ricco’s area in the non-amblyopic eyes of binocular amblyopes compared to those of non-binocular amblyopes and normal controls. As evident in Fig. 4, it can be seen that when Ricco’s area estimates were divided into binocular and non-binocular groups, mean values in the non-amblyopic eyes of the non-binocular group closely resemble those of normal controls. In fact, it is those estimates in the non-amblyopic eyes of the binocular group that are smaller than normal. This finding could be explained by a simple cortical model similar to that proposed by McKee *et al*. (their Appendix A)^55^. Suppose that in a given region of the visual cortex of a binocular amblyope, 60% of neurons are binocularly-driven (i.e. they receive input from both eyes), and the remaining 40% of neurons are monocularly-driven (20% from each eye). Then, suppose that in non-binocular amblyopes, the same region of the visual cortex contains only monocularly-driven neurons; 50% receiving input from one eye and the other 50% receiving input from the fellow eye. Full-field monocular stimulation in binocular amblyopes would result in stimulation of up to 80% of cortical cells in that region. In non-binocular amblyopes, the same degree of monocular stimulation would elicit a response of up to 50% of cortical cells in that region. If a stimulus of a fixed area is projected onto the retina of one eye, the number of responding RGCs should be equal in both groups (assuming no retinal stretching). However, if the number of cortical cells responding to the stimulus is smaller in the non-binocular group, Ricco’s area may be larger because of greater convergence of signals from the same number of geniculocortical axons on to a smaller cortical region. Conversely, in the binocular group, Ricco’s area may be smaller than that in the non-binocular group, because of less convergence of signals from the same number of geniculocortical axons on to a larger cortical region. Swanson *et al*. determined that Ricco’s area is sampled by a critical number of RGCs (n = 31) across the visual field of a normal observer under perimetric conditions equivalent to those employed in our study^36^. If one assumes that Ricco’s area is also sampled by a critical number of cortical cells across the visual field, a monocularly-presented stimulus of a fixed area would be sampled by approximately 16% more cortical cells in a given cortical region in binocular amblyopes than in non-binocular amblyopes. In this case, the critical number of cortical cells, and thus the criterion for the extent of Ricco’s area, would be met with a smaller stimulus, resulting in a smaller Ricco’s area in those eyes.

The results of our study also have important implications for our understanding of visual field sensitivity deficits in glaucoma. Attempts to understand the nature of sensitivity loss in glaucoma have typically involved investigations of the relationship between RGC number (or a surrogate) and visual field sensitivity to achromatic circular luminance increments on a uniform achromatic background (conventional perimetry). Guided by the fact that glaucoma is characterised by death of RGCs, many investigations do not consider changes that may occur at extra-retinal levels, but instead make decisions on the utility of one functional test over another based on the strength of association between the test output and measurements of retinal structure. Given that an enlarged Ricco’s area is also observed in glaucoma^48^, the results of our study provide further support for the case that changes in cortical mechanisms should be taken into account when attempting to understand the nature of visual loss in glaucoma, measured with conventional circular incremental stimuli. A more in-depth discussion of this issue is given by Rountree *et al*.^68^.

In conclusion, a larger-than-normal Ricco’s area has been found in amblyopic eyes, and a smaller-than-normal area has been found in fellow non-amblyopic eyes in our sample of participants. This finding suggests that Ricco’s area is the psychophysical consequence of multiple pooling mechanisms in the visual cortex, rather than in retinal receptive fields alone. Greater attention should therefore be given to alterations in cortical processing in glaucoma, given that a loss of sensitivity to conventional stimuli can be mapped to an enlarged Ricco’s area. The findings in the current study also highlight differences in fundamental attributes of visual function between binocular and non-binocular amblyopes as well as strabismic and anisometropic amblyopes that warrant further investigation.
